# My Tribute to Mary Ellen Avery

**DOI:** 10.3389/fped.2014.00031

**Published:** 2014-04-23

**Authors:** John Steven Torday

**Affiliations:** ^1^Department of Pediatrics, University of California Los Angeles, Los Angeles, CA, USA

**Keywords:** lung surfactant, soap bubbles, Boston Lying-In Hospital, joint program in neonatology, Montreal Chidren’s Hospital, Boston Children’s Hospital, Brigham and Women’s Hospital, antenatal steroids

“Life is that which can mix oil and water”

*– Robert Frost*

Mary Ellen Avery was a force of Nature.

I first met her while I was a graduate student at McGill University in the early 70’s. She and her colleagues had been studying the effects of glucocorticoids on fetal lung maturation and surfactant production, and had stumbled onto a curious “neighbor effect” – when they treated one of the fetal rabbits in the womb by direct injection, they found an effect on the maturation of the lungs in the collateral pups. I was studying fetal endocrinology at the time in Claude Giroud’s Laboratory at Montreal Children’s Hospital, and had access to radiolabeled cortisol, so I could determine if the hormone was passing from one fetus to the other. Having demonstrated this effect, Dr. Avery named me as a co-author on their paper describing this phenomenon, which was quite generous of her – but that was in her nature, as I was to discover in a 20-year journey with her as my mentor.

In the spring of 1974, while on an elevator at Montreal Children’s Hospital, Dr. Avery invited me to join her research group at Harvard Medical School. She said that it was important to maintain the highest scientific standards in developing the burgeoning discipline of Neonatology because she was concerned about doing harm in the name of doing good. I had already committed to a post-doctoral position with Jack Gorski and N.L. First in the NIH Reproductive Endocrine Program at the University of Wisconsin-Madison, so I had to decline the invitation, with the understanding that I would come to Boston after my Fellowship. I joined the Joint Program in Neonatology in July, 1976 as a Research Associate. During my early years at the Boston Lying-In Hospital (BLI), where in the 1950s Dr. Avery had previously discovered that Hyaline Membrane Disease was due to surfactant deficiency in preterm newborns, I conducted studies on the physiologic role of hormones in fetal lung development in support of the clinical use of antenatal steroids to accelerate the production of surfactant. Antenatal steroid treatment dramatically improved the survival of preterm infants. It is considered one of the major breakthroughs of twentieth century medicine, saving the lives of hundreds of thousands of newborns. During the course of the first clinical trials of antenatal steroids for the prevention of Respiratory Distress Syndrome, it was found that males were much less responsive to such treatment than females, reprising my interest in the sexual dimorphism of fetal development, the subject of my Masters’ thesis in graduate school at McGill. As always, Dr. Avery was open to whatever we wanted to study, so one of her Neonatal Fellows, Heber Nielsen, and I began a 20-year investigation of this mechanism in trying to maximize the benefit of antenatal steroid therapy.

During that era, the Director of the Joint Program in Neonatology, H. William Taeusch, asked me to start a clinical laboratory for the measurement of lung surfactant at the BLI. Since this was a direct extension of my basic scientific work, I accepted the challenge. The laboratory began processing amniotic fluid samples in the spring of 1977 in parallel with the advent of the clinical implementation of antenatal steroids. At that time, the standard method for measuring surfactant was the L/S ratio, which was known to lack sensitivity and specificity, but prior to the implementation of steroid therapy the management of preterm birth was essentially passive, so that method was adequate. But with the onset of the use of antenatal steroids, there was a sea change in Neonatology, the fetus becoming a patient treated in the womb. To improve on the antenatal testing for lung maturation, I developed the Saturated Phosphatidylcholine Assay, which was far superior to the L/S Ratio, being more than 90% accurate in predicting the risk of Respiratory Distress Syndrome. So here was an example of how the burgeoning discipline of Neonatology was able to use experimental methods to optimize the well-being of preterm newborns.

During that era, we used to have 4th of July picnics for the Joint Program in Neonatology. We’d inevitably have to play softball because it was Dr. Avery’s passion. She loved to pitch. I was sitting on the sidelines with my then 2-year old daughter, who turned to me at one point and said loud enough so all could hear, “Dr. Avery doesn’t do that very well.” It was then and there that I knew I would not live out my days at Harvard.

Meanwhile, our basic research effort to understand why males were refractory to antenatal glucocorticoid treatment was advancing. We were able to determine that this was due to a physiologic mechanism by which androgens delayed the maturation of the fetal lung, allowing for persistence of the growth phase of lung development. This was due to androgen perpetuating the production of Transforming Growth Factor Beta in the connective tissue cells surrounding the alveoli, promoting more, but immature alveoli.

I left Boston in 1991, joining the Neonatal Division at the University of Maryland. It was there that I discovered the Neutral Lipid Trafficking phenomenon – the active movement of lipid substrate between connective tissue and epithelial cells mediated by specific signaling mechanisms stimulated by both hormones and mechanical stretch. Dr. Avery had kept in touch with me, and knew of my interest in the role of “stretch” in lung development, sending me scientific papers to keep me on-track. The annual meeting of the Society for Pediatric Research was held in Baltimore in 1992, so while attending the meeting Dr. Avery paid me a visit in my new laboratory. She handed me a book, entitled *Soap Bubbles: Their Colors and Forces Which Mold Them*, by C.V. Boys, saying that everything she knew about lung surfactant was in that book! Telling me in her own way to keep it simple (KISS). In fact her perennial question to her students regarding whether something was worth studying was “can you live without it?”

Further study of the cellular–molecular signaling mechanisms for alveolar surfactant homeostasis have led to a fundamental understanding of how physiology has evolved. Now there is a way of understanding the how and why of physiology from its origins in unicellular organisms, providing a “logic” that simplifies what had become artificially complicated. I am indebted to Mary Ellen Avery for giving me the opportunity to pursue new knowledge.

She is missed.

## Conflict of Interest Statement

The author declares that the research was conducted in the absence of any commercial or financial relationships that could be construed as a potential conflict of interest.

